# Naringenin Targets PI3K p85alpha to Suppress PI3K/AKT Signaling Pathway and Ameliorate Disordered MMP-9 Secretion in Cigarette Smoke Extract-Induced Alveolar Macrophages In Vitro

**DOI:** 10.3390/cells14100678

**Published:** 2025-05-08

**Authors:** Weiyang Fan, Ziyan Xu, Mengli Zhong, Xiao Wu, Pan Chen, Zhen Chen, Weiwei Su, Hao Wu, Peibo Li

**Affiliations:** Guangdong Provincial Key Laboratory of Plant Stress Biology, State Key Laboratory of Biocontrol, Guangdong Engineering and Technology Research Center for Quality and Efficacy Re-Evaluation of Post Marketed TCM, School of Life Sciences, Sun Yat-sen University, Guangzhou 510275, China; fanwy5@mail2.sysu.edu.cn (W.F.); xuzy39@mail2.sysu.edu.cn (Z.X.); zhongmli@mail2.sysu.edu.cn (M.Z.); wuxiao53@mail2.sysu.edu.cn (X.W.); chenpan989@126.com (P.C.); chenzh358@mail2.sysu.edu.cn (Z.C.); lsssww@mail.sysu.edu.cn (W.S.)

**Keywords:** alveolar macrophage, cigarette, MMP-9, naringenin, PI3K p85alpha

## Abstract

Background: Naringenin has demonstrated potential therapeutic effects against cigarette smoke-induced lung injury; however, its underlying mechanisms of regulating matrix metalloproteinase-9 (MMP-9) in alveolar macrophages remain unclear. Methods: The regulatory mechanisms of naringenin in cigarette smoke extract (CSE)-induced alveolar macrophages were investigated using proteomics, and then, naringenin’s targets were further validated by Western blot, molecular docking, molecular dynamics (MD) simulations, cellular thermal shift assay (CETSA), and enzyme activity assay. Results: The proteomics revealed that the PI3K/AKT signaling pathway might play a crucial role in naringenin’s inhibition of MMP-9. Western blot analysis confirmed that naringenin significantly inhibited CSE-upregulated PI3K/AKT signaling pathway and reduced MMP-9 expression in MH-S cells. Notably, the PI3K activator 740Y-P reversed naringenin’s effects on MMP-9. Additionally, molecular docking, MD simulations, and CETSA identified PI3K p85alpha as the potential binding site for naringenin, and naringenin markedly inhibited CSE-induced PI3K activity. In in vitro experiments, naringenin inhibiting MMP-9 secretion in alveolar macrophages contributed to alleviating elastin and E-cadherin damage in alveolar epithelial cells. Furthermore, naringenin effectively suppressed CSE-induced MMP-9 secretion in primary mouse alveolar macrophages and human THP-1-differentiated macrophages. Conclusions: Our findings revealed that naringenin, a potential candidate for treating smoking-induced lung injury, directly targeted PI3K p85alpha, inhibiting PI3K activity and MMP-9 expression in CSE-induced alveolar macrophages via suppressing the PI3K/AKT signaling pathway.

## 1. Introduction

Smoking still remains a major public health challenge [1]. Cigarette smoke is a complex and dynamic chemical mixture containing thousands of substances, including nicotine, tar, carbon monoxide, nitrogen oxides, carbonyl compounds, aromatic amines, polycyclic aromatic hydrocarbons, nitrosamines, and toxic heavy metals [2,3]. Many of the substances in cigarette smoke are cytotoxic, and at least 20 of these are recognized carcinogens [4]. Prolonged exposure to cigarette smoke significantly increases the risk of developing lung diseases such as chronic obstructive pulmonary disease (COPD), asthma, emphysema, and pulmonary fibrosis, posing a severe threat to human health [5,6]. Studies indicated that in 2020, approximately 1.18 billion people worldwide were smokers, and 177 million suffered from disabilities attributable to smoking [7]. According to a report from the World Health Organization in 2023, over 8 million people died prematurely each year due to smoking, with more than 7 million deaths resulting from direct smoking and around 1.2 million from secondhand smoke exposure [8].

Alveolar macrophages are the primary immune cells in the lungs and constitute over 90% of lung macrophages in homeostasis [9]. Alveolar macrophages are widely distributed on the surface of airways and alveoli, directly exposed to the external environment [10]. They are among the first cells to interact with foreign substances and serve as a critical defense line against inhaled particulates and pathogens [11]. Various functions of alveolar macrophages, including recruitment, phenotype, immune responses, and intracellular homeostasis, have been shown to be dysregulated after exposure to cigarette smoke [12]. Studies had demonstrated that cigarette smoke disrupted the oxidative and antioxidant balance in the lungs, induced DNA damage and cell cycle arrest, and stimulated alveolar macrophages to secrete pro-inflammatory cytokines and chemokines, leading to lung inflammation and parenchymal injury [13].

Matrix metalloproteinases (MMPs) are a highly conserved family of proteases involved in various biological processes such as airway development, wound healing, angiogenesis, and cell migration [14]. Alveolar macrophages secrete MMPs to remodel the extracellular matrix (ECM) [15], contributing to the development of airflow limitation and playing a key role in the progression of diseases like COPD, emphysema, pulmonary fibrosis, and acute respiratory distress syndrome [16,17]. Matrix metalloproteinase-9 (MMP-9), also known as gelatinase B, is a member of the MMP family and is primarily responsible for ECM degradation and remodeling [18]. Research has indicated that MMP-9 is crucial in immune regulation and the development of lung inflammation-related diseases [19,20]. Alveolar macrophages were the primary source of MMP-9 in the lungs of patients with emphysema [21], and its overproduction led to the loss of alveolar elastin and irreversible alveolar destruction [22]. The increased expression of MMP-9 elevated the risk of cigarette smoke-induced emphysema [23]. Therefore, inhibiting MMP-9 secretion from alveolar macrophages is a potential therapeutic strategy for lung injury in smokers, and it remains a subject of ongoing investigation.

Naringenin, a natural dihydroflavonoid compound (4′,5,7-trihydroxyflavanone) widely presented in citrus fruits, exhibits anti-inflammatory and antioxidant pharmacological activities [24]. Naringenin can directly scavenge intracellular free radicals, enhance the endogenous antioxidant defense system, and inhibit the expression of various inflammation-related transcription factors and pro-inflammatory cytokines [25,26]. Pulmonary delivery of naringenin has been explored to overcome the low oral bioavailability, and naringenin showed better efficacy via aerosol inhalation with potential for clinical application in treating inflammatory lung diseases [27,28]. MMP-9 suppression by naringenin has been extensively documented in animal models of lung injury [29] and other inflammatory diseases [30,31]. Although studies have shown that naringenin dose-dependently suppressed MMP-9 expression in pulmonary epithelial cells [32], the regulatory mechanisms of naringenin in alveolar macrophages remains unclear.

In this study, we evaluated the effects of naringenin on cigarette smoke extract (CSE)-induced MMP-9 expression and secretion in MH-S cells. Using proteomics analysis, molecular docking, MD simulations, and cellular thermal shift assay (CETSA), we investigated the regulatory mechanisms by which naringenin inhibits CSE-induced MMP-9 expression through suppressing the PI3K/AKT signaling pathway. Our results demonstrated that naringenin directly bound to PI3K p85alpha, enhancing the stability of its phosphorylated protein and inhibiting PI3K activity. The reduced secretion of MMP-9 from CSE-exposed alveolar macrophages was conducive to protecting alveolar epithelial elastin and E-cadherin from damage. These findings provided a supplemented pharmacological basis for naringenin as a potential treatment for cigarette smoke-induced lung injury.

## 2. Materials and Methods

### 2.1. Chemicals and Reagents

CSE was prepared according to our previous report, using the smoke extract from Cocopalm cigarettes (6901028002011, Guangzhou, China) with consistent quality control [33,34]. The naringenin (C_15_H_12_O_5_, CAS: 67604-48-2) was purchased from Sigma-Aldrich (Milwaukee, WI, USA) with a purity of 98%. MH-S cells (mouse alveolar macrophage cell line, CRL-2019), MLE-12 cells (mouse alveolar epithelial cell line, CRL-2110), and THP-1 cells (human monocytic leukemia cell line, TIB-202) were purchased from American Type Culture Collection (Rockville, MD, USA). 740Y-P (CSN19217) was purchased from CSNpharm (Chicago, IL, USA). Dimethyl sulfoxide (DMSO, HY-Y0320) and phorbol 12-myristate 13-acetate (PMA, HY-18739) were purchased from MedChemExpress (New Jersey, NJ, USA).

### 2.2. Cell Culture and Treatment

MH-S cells were cultured in RPMI-1640 medium (C11875500BT, Gibco, Carlsbad, CA, USA) supplemented with 0.05 mM 2-mercaptoethanol. MLE-12 cells, THP-1 cells, and primary mouse alveolar macrophages were maintained in RPMI-1640 medium. The base media were supplemented with 10% fetal bovine serum (CM1002S, Cellcook, Guangzhou, China), and cells were grown at 37 °C with 5% CO_2_. Before the experiment, THP-1 cells were differentiated into macrophages using 100 ng/mL PMA for 12 h treatment.

MH-S cells, differentiated THP-1 cells, or primary mouse alveolar macrophages were divided into groups and treated for 24 h: blank control; model group (2.5% CSE); vehicle control (2.5% CSE + 0.1% DMSO); co-treatment groups with 2.5% CSE and varying naringenin concentrations (10, 30, 100, or 300 μM); and 2.5% CSE + 100 μM naringenin + 30 μM 740Y-P (PI3K activator). Post-treatment, samples were collected for downstream assays. For MLE-12 cells, supernatants from MH-S cells (after 24 h treatment) were removed, followed by sterile PBS washing and fresh medium replacement. After 12 h of additional culture, conditioned medium (CM) from corresponding MH-S cell groups was collected and co-incubated with MLE-12 cells for 24 h prior to analysis. Additionally, corresponding CM was supplemented with either an anti-MMP-9 antibody (100 ng/mL, ab283594, Abcam, Cambridge, UK) or isotype-matched IgG control (100 ng/mL, ab172730, Abcam, Cambridge, UK) for 24 h to verify MMP-9 roles.

### 2.3. Cell Viability Assay

Cell viability of alveolar macrophages was measured by MTS solution (G3580, Promega, Madison, WI, USA). Briefly, MH-S cells were grown in 96-well plates with different treatments. After processing, 10% MTS solution was added and incubated for 1 h. The absorbance of 490 nm was assayed by the Epoch microplate reader (BioTek, Winooski, VT, USA).

### 2.4. Enzyme Linked Immunosorbent Assay (ELISA)

ELISA kits for MMP-9 (SEA553Mu and SEA553Hu, Cloud-Clone, Wuhan, China), elastin (JL26168, JonlnBio, Shanghai, China), and E-cadherin (JL41795, JonlnBio, Shanghai, China) were used to measure the cytokine levels in the cells or supernatants. In brief, the collected supernatants or the lysed cells were centrifuged at 2000× *g* for 10 min and then were utilized for subsequent ELISA measurement. Following the manufacturer’s protocols, the 450 nm absorbance was recorded on the Epoch microplate reader.

### 2.5. Proteomics Analysis

Label-free data-independent acquisition (DIA) for proteomics was performed. In brief, proteins from MH-S cells were extracted via cryogenic grinding with 8 M Urea, 50 mM Tris-HCL, and EDTA-free Protease Inhibitor Cocktail (5892791001, Roche, Basel, Switzerland). Following protein collection, samples were reduced with 10 mM dithiothreitol and alkylated with 20 mM iodoacetamide to disrupt disulfide bonds and prevent re-oxidation. The protein concentration was quantified using the Bradford Protein Assay Kit (P0006, Beyotime, Shanghai, China). Sodium dodecyl sulfate-polyacrylamide gel electrophoresis (SDS-PAGE) was employed to observe protein molecular weights. Post-enzymatic digestion using 3 μg trypsin, the 150 μg proteins were desalted using Waters solid phase extraction cartridges and then vacuum-dried.

Samples for three groups (treatment for 24 h: control; 2.5% CSE; 2.5% CSE + 100 μM naringenin. *n* = 5) were redissolved with 0.1% formic acid and analyzed using Q Exactive HF^TM^ LC-MS (Thermo Fisher Scientific, San Jose, CA, USA). In short, samples underwent gradient separation on a C18 column (100 μm internal diameter, 1.8 μm particle size, 35 cm length) in liquid chromatography (LC) at a flow rate of 300 nL/min: 0~103 min, mobile phase B (mobile phase A: 0.1% formic acid; mobile phase B: 0.1% formic acid and 98% acetonitrile) linearly rose from 4% to 27%; 103~111 min, mobile phase B increased from 27% to 40%; 111~113 min, mobile phase B increased from 40% to 90%; 113~120 min, 90% mobile phase B. The separated samples were ionized by nano-ESI and then transferred to a mass spectrometer (MS) for DIA mode detection. Primary MS scan (350–1500 *m*/*z*) was acquired at 120,000 resolution with a 300% automatic gain control target. For DIA, 53 variable isolation windows (400–1200 *m*/*z*) were applied. Higher-energy collision dissociation fragmentation at 32% energy was followed by fragment ion detection in the Orbitrap (30,000 resolution; 200% automatic gain control target).

The DIA-NN version 1.8 was used for protein identification and quantification [35]. Data were searched against protein sequences from the UniProt database with the following settings: Trypsin/P, max 2 missed cleavages, fixed carbamidomethylation (C), variable oxidation (M), variable N-terminal acetylation, precursor mass tolerance of 20 ppm, and 0.05 Da fragment mass tolerance. The results were filtered by 1% false discovery rate (FDR). The significance threshold *p*-value was 0.05 (Benjamini–Hochberg FDR < 0.1 for all), and the fold change (FC) was greater than 1.2 or less than 0.83 in each test (ProteomeXchange: PXD063137), while differential protein analysis was conducted using R version 4.0 (R Foundation for Statistical Computing, Vienna, Austria).

### 2.6. Western Blot Analysis

MH-S cells after different treatments were disintegrated on ice with RIPA Lysis Buffer (P0013B, Beyotime, Shanghai, China) supplemented with EDTA-free Protease Inhibitor Cocktail. The obtained lysates were subjected to centrifugation at 12,000× *g* for 30 min at 4 °C, and then, the collected supernatants were quantified by BCA Protein Assay Kit (P0010S, Beyotime, Shanghai, China). Subsequently, the concentration-normalized proteins were denatured with SDS-PAGE Sample Loading Buffer (P0015L, Beyotime, Shanghai, China) by heating at 95 °C for 10 min. According to the manufacturer’s protocols, the samples (10 μg proteins per blot) were separated with the 7.5% TGX FastCast Kit (1610171, Bio-Rad, Hercules, CA, USA) and transferred onto polyvinylidene fluoride (PVDF) membranes using the Trans-Blot Turbo RTA Mini PVDF Transfer Kit (1704272, Bio-Rad, Hercules, CA, USA). The primary antibodies against phosphoinositide 3-kinase p110beta (PI3K p110beta, 1:1000 dilution, 20584-1-AP, Proteintech, Wuhan, China), PI3K p85alpha (1:5000 dilution, 60225-1-Ig, Proteintech, Wuhan, China), phospho-PI3K p85alpha (Y467) (p-PI3K p85alpha, 1:1000 dilution, ab278545, Abcam, Cambridge, UK), protein kinase B (AKT, 1:2000 dilution, ab179463, Abcam, Cambridge, UK), phospho-AKT1 (pS473) + AKT2 (pS474) + AKT3 (pS472) (p-AKT, 1:1000 dilution, ab192623, Abcam, Cambridge, UK), MMP-9 (1:1000 dilution, ab38898, Abcam, Cambridge, UK), and vinculin (1:2500 dilution, ab129002, Abcam, Cambridge, UK) were used as recommended by the manufacturer. After incubation, the PVDF membranes were treated with corresponding HRP-conjugated secondary antibodies (W4011 or W4021, 1:5000 dilution, Promega, Madison, WI, USA). The signals were visualized by Tanon5200 (Tanon, Shanghai, China) in accordance with Clarity Western ECL Subetrate (1705061, Bio-Rad, Hercules, CA, USA), and the densities were analyzed by imageJ (National Institutes of Health, Bethesda, MD, USA).

### 2.7. Molecular Docking Studies

The 3D molecular structure of naringenin (PubChem CID: 439246) was obtained from PubChem (https://pubchem.ncbi.nlm.nih.gov, accessed on 9 May 2024). The 3D protein structures of PI3K p110beta (UniProtKB Entry: Q8BTI9), PI3K p85alpha (UniProtKB Entry: P26450), AKT1 (UniProtKB Entry: P31750), AKT2 (UniProtKB Entry: Q60823), AKT3 (UniProtKB Entry: Q9WUA6), and MMP-9 (UniProtKB Entry: P41245) were acquired from the AlphaFold Protein Structure Database (https://alphafold.ebi.ac.uk, accessed on 9 May 2024), a high-accuracy prediction database (manually annotated and reviewed via UniProt reference proteomes). As previously reported [36], molecular docking was, respectively, performed in 9 docking runs using BatchVinaGUI v2.2.0. A default minimum grid box size of 2.00 Å was used for blind docking and adjusted during the autodock process. The final binding conformations were visualized by PyMOL v2.5.0.

### 2.8. Molecular Dynamics (MD) Simulations

MD simulations were executed using Gromacs 2019.6, with a total simulation duration of 100 ns [37]. In brief, the protein and ligand were subjected to the Amber14SB force field and the TIP3P water model, which were encapsulated in a periodic cubic box 0.1 nm from the boundary, filled with n-decane, water molecules, and neutralized counterions. The equilibration process utilized the NVT ensemble, followed by production simulations with the NPT ensemble for 100 ps at a temperature of 310.15 K. Finally, 100 ns trajectory analyses were conducted, including secondary structure, root mean square deviation (RMSD), root mean square fluctuation (RMSF), radius of gyration (Rg), solvent accessible surface area (SASA), hydrogen bond (H-bond), free energy landscape (FEL), and molecular mechanics Poisson–Boltzmann surface area (MMPBSA).

### 2.9. Cellular Thermal Shift Assay (CETSA)

CETSA was performed as previously described [38]. MH-S cell lysates (2 mg/mL) were prepared and incubated with 1 nM naringenin or equal volume of DMSO for 1 h at 4 °C. Subsequently, the lysates were divided into aliquots and heated, respectively, at different temperatures (45, 50, 55, 60, 65, 70 °C) for 10 min using a thermal cycler, followed by cooling at 4 °C. The supernatants were collected after centrifugation at 20,000× *g* for 20 min at 4 °C and then subjected to Western blot analysis.

### 2.10. Determination of Class/PI3K Activity

The class I PI3K activity was directly measured through its reaction product, phosphatidylinositol 3,4,5-triphosphate (PIP3), using a PI3K Profiling Kit (K-1000s, Echelon Biosciences lnc., Salt Lake City, UT, USA). Briefly, the processed samples underwent the PI3K enzyme reaction, followed by a 1 h incubation with PIP3-binding proteins in a microplate. The samples were then transferred to a coated 96-well plate for a 1 h competitive binding incubation. After three washes, secondary antibodies were added and incubated for 30 min, followed by three more washes. TMB substrates were added for a 30 min incubation in the dark, and the absorbance of 450 nm was detected after adding stop solution.

### 2.11. Primary Mouse Alveolar Macrophages Preparation and Marker Staining

Primary mouse alveolar macrophages were isolated from bronchoalveolar lavage fluid (BALF) in 6–8-week-old male C57BL/6 mice, and the C57BL/6 mice were purchased from the Laboratory Animal Center, Sun Yat-sen University (number of animal use permit: SYXK 2023-0112). On the basis of reported protocols, the anesthetized mice were euthanized by cervical dislocation, followed by BALF collection [39]. After centrifugation at 500× *g* for 5 min at 4 °C, the cell pellets of each mouse were resuspended in 0.5 mL RPMI-1640 medium and cultured in 24-well plates. The first medium exchange was conducted after 1.5 h culture, and the adherent cells were retained for the next experiment or in vitro cultivation. To characterize whether the adherent cells obtained from BALF were alveolar macrophages, PE-conjugated CD11b antibody (101207, BioLegend, San Diego, CA, USA) and Alexa Fluor 647-conjugated F4/80 antibody (123121, BioLegend, San Diego, CA, USA) were used to detect macrophage markers after FcR blocking by CD16/32 antibody (101319, BioLegend, San Diego, CA, USA). Images were captured using an SP8X laser scanning confocal microscope (Leica, Wetzlar, Germany).

### 2.12. Statistical Analysis

Scientific illustrations were created with BioRender (https://www.biorender.com, accessed on 20 December 2024). The statistical analysis and graphing were performed using GraphPad Prism 8 (La Jolla, CA, USA), with results expressed as the mean ± standard deviation (SD). Unless otherwise specified, experiments were independently conducted with at least six repetitions (*n* = 6) and normalized to the control group when necessary. Statistical comparisons between two groups were performed using the unpaired two-tailed *t* test, while multi-group comparisons were analyzed using ordinary one-way ANOVA with Dunnett’s test. Values of * *p* < 0.05 and ** *p* < 0.01 were considered statistically significant.

## 3. Results

### 3.1. Naringenin Inhibited the Secretion of MMP-9 in CSE-Induced MH-S Cells

**Figure 1:**
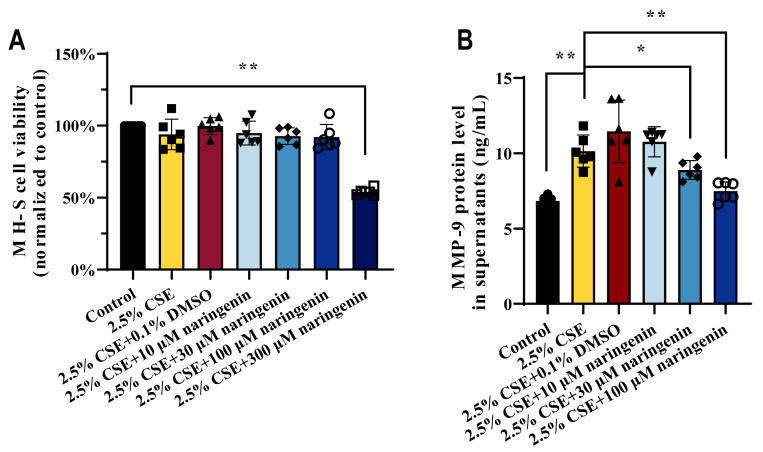
Naringenin diminished MMP-9 secretion in CSE-induced MH-S cells. The effects of CSE and naringenin for 24 h treatment on (**A**) cell viability and (**B**) level of MMP-9 secreted in MH-S cells. Data are expressed as mean ± SD (*n* = 6). * *p* < 0.05, ** *p* < 0.01.

Naringenin, a natural trihydroxy flavanonide with low toxicity and high efficacy, exhibits anti-inflammatory and immunomodulatory properties [40]. In a previous study, we performed CSE at concentrations of 2.5%, 5%, 7.5%, 10%, 12.5%, and 15% for 24 h treatment in MH-S cells, where 2.5% CSE did not impair alveolar macrophage viability [33]. Here, we chose 2.5% CSE concentration for subsequent studies to evaluate the potential effects on MMP-9 when subjected to naringenin treatment. Our findings illustrated in Figure 1A showed that the 2.5% CSE-stimulated MH-S cell viability remained largely unaffected by naringenin concentrations up to 100 μM. Therefore, the naringenin doses of 10, 30, and 100 μM were selected for subsequent experiments. Figure 1B reveals that 2.5% CSE significantly increased the secretion of MMP-9 in MH-S cell supernatants compared with the control group, while 30 μM and 100 μM naringenin markedly decreased the CSE-stimulated MMP-9 secretion.

### 3.2. Proteomics Analysis of Naringenin Treatment in CSE-Induced MH-S Cells

**Figure 2:**
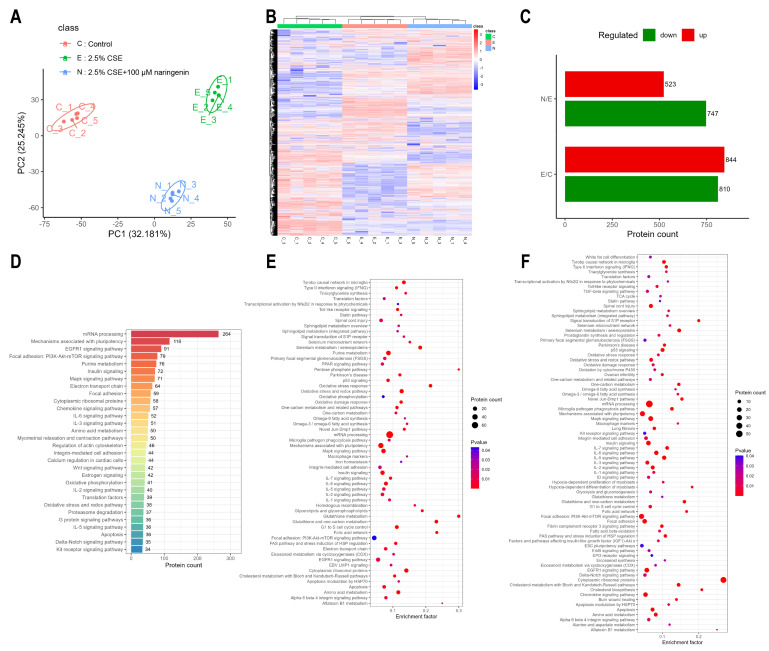
The PI3K/AKT signaling pathway was identified as a significantly altered target through proteomics analysis. (**A**) Principal component analysis, (**B**) heat map, (**C**) differentially expressed proteins, and (**D**) the top 30 pathway annotations by WikiPathways in identified proteins of the three groups. WikiPathways enrichment analysis of differentially expressed proteins: (**E**) 2.5% CSE group vs. control group; (**F**) 2.5% CSE + 100 μM naringenin group vs. 2.5% CSE group. Data are presented with *n* = 5. The results were filtered by 1% FDR. The differential proteins among groups were accepted when the *p*-value was less than 0.05 and FC was greater than 1.2 (upregulation) or less than 0.83 (downregulation).

In order to evaluate the regulatory mechanism of naringenin on MMP-9 expression, MH-S cells were treated with 100 μM naringenin in the presence of 2.5% CSE for 24 h, and then, the quantitative proteomic profile was depicted. PCA and heat map show that the naringenin treatment group exhibited a very distinct protein expression pattern compared with that of the control and the CSE groups (Figure 2A,B). The analysis identified 844 upregulated and 810 downregulated proteins in the CSE group compared with the control group, as well as 523 upregulated and 747 downregulated proteins in the naringenin treatment group compared with the CSE group (Figure 2C). The WikiPathways enrichment analysis of these identified proteins suggested their involvement in mRNA processing, pluripotency mechanism, the EGFR1 signaling pathway, and the PI3K/AKT signaling pathway (Figure 2D). These differentially expressed proteins were compared to identify signaling pathways associated with MMP-9 (Figure 2E,F). It was noteworthy that the PI3K/AKT signaling pathway, as one of the regulators of MMP-9, was upregulated in the CSE group and downregulated by co-treatment with naringenin, which may explain the inhibition of CSE-induced MMP-9 secretion in MH-S cells by naringenin.

### 3.3. Naringenin Decreased CSE-Induced Expressions of the PI3K/AKT Signaling Pathway in MH-S Cells

**Figure 3:**
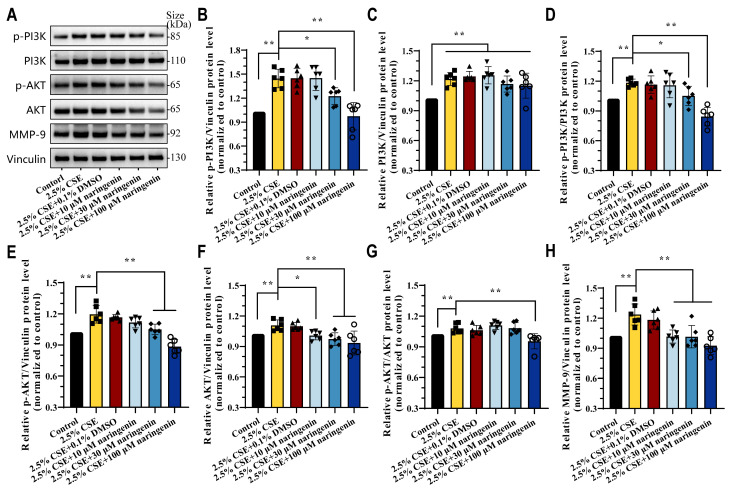
Naringenin inhibited CSE-induced PI3K/AKT/MMP-9 signaling pathway. (**A**) Representative images and (**B**–**H**) relative protein expression levels of (**B**) p-PI3K p85alpha, (**C**) PI3K p110beta, (**D**) p-PI3K p85alpha/PI3K p110beta, (**E**) p-AKT, (**F**) AKT, (**G**) p-AKT/AKT, and (**H**) MMP-9 in CSE-induced MH-S cells treated with naringenin for 24 h by Western blot. Data are expressed as mean ± SD (*n* = 6). * *p* < 0.05, ** *p* < 0.01.

Accumulating evidence indicate the PI3K/AKT signaling pathway played a key role in CSE-induced MMP-9 expression. Therefore, we performed Western blot analysis to investigate the effects of naringenin on the PI3K/AKT signaling pathway in CSE-induced MH-S cells after 24 h treatment. Figure 3 illustrates the pronounced induction of p-PI3K, PI3K, p-AKT, AKT, and MMP-9 expression following 2.5% CSE stimulation. Significantly, 2.5% CSE increased the ratio of p-PI3K/PI3K and p-AKT/AKT protein levels compared to the control group. In contrast, the intervention of 30 μM and 100 μM naringenin reduced this elevation in the relative protein levels of p-PI3K, p-AKT, AKT, and MMP-9, while 100 μM naringenin effectively attenuated the effects of CSE induction on p-PI3K/PI3K and p-AKT/AKT, and these results showed a consistent tendency with the proteomic analysis.

### 3.4. Naringenin Inhibited CSE-Induced MMP-9 Expression and Secretion by Downregulation of PI3K Phosphorylation

**Figure 4:**
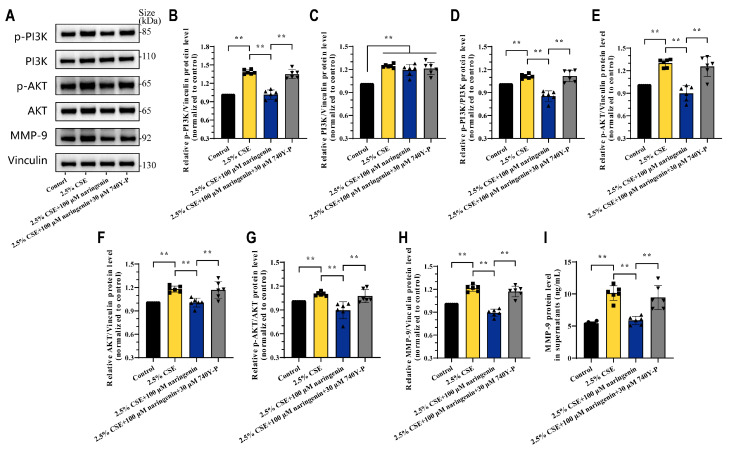
Activation of PI3K phosphorylation neutralized the beneficial effects of naringenin against CSE-induced MMP-9 expression. (**A**) Representative images and (**B**–**H**) quantification for the protein levels of (**B**) p-PI3K p85alpha, (**C**) PI3K p110beta, (**D**) p-PI3K p85alpha/PI3K p110beta, (**E**) p-AKT, (**F**) AKT, (**G**) p-AKT/AKT, and (**H**) MMP-9 at 24 h treatment by Western blotting. (**I**) Quantitative protein secretion levels of MMP-9 in MH-S cell supernatants at 24 h treatment by ELISA. Data are expressed as mean ± SD (*n* = 6). ** *p* < 0.01.

740 Y-P is a powerful, cell-permeable agonist of PI3K and is widely used to study the PI3K/AKT signaling pathway [41]. To further examine whether naringenin regulated the PI3K/AKT axis to inhibit CSE-induced MMP-9, MH-S cells were treated with 740Y-P for 24 h in the presence of 2.5% CSE and naringenin. Interestingly, 30 μM 740Y-P restored the expression of p-PI3K, p-PI3K/PI3K, p-AKT, AKT, and p-AKT/AKT in MH-S cells that were downregulated by 2.5% CSE and 100 μM naringenin treatment, and it also reversed MMP-9 expression and secretion (Figure 4). Above all, our results underscored the pivotal role of PI3K/AKT in MMP-9 expression and revealed the interplay of CSE and naringenin in MH-S cells. While naringenin did not directly influence PI3K protein expression, it emerged as a potent regulator in suppressing the PI3K phosphorylation in macrophages.

### 3.5. Molecular Docking Studies Between Naringenin and PI3K/AKT/MMP-9 Proteins

**Figure 5:**
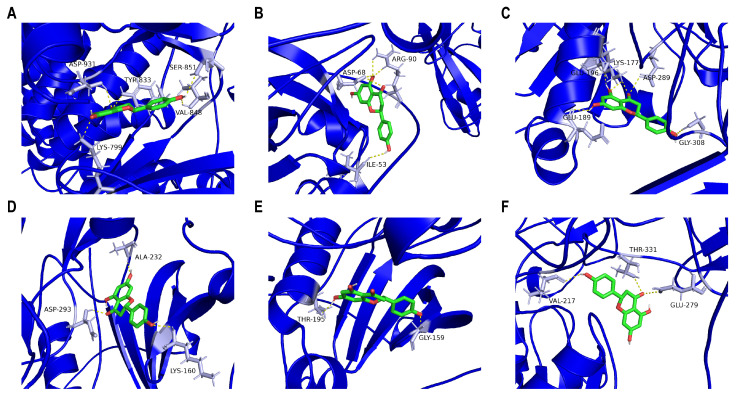
Molecular docking validation. Ligand–receptor docking revealed that naringenin could bind to (**A**) PI3K p110beta, (**B**) PI3K p85alpha, (**C**) AKT3, (**D**) AKT2, (**E**) AKT1, or (**F**) MMP-9 through amino acid residues. The 3D molecular structure of naringenin is in green, and the 3D protein structure of the corresponding target protein is in blue. The yellow dotted line signifies a potential bond, and the corresponding amino acid residues are in grey.

To evaluate the targets of naringenin in modulating PI3K/AKT/MMP-9, we carried out molecular docking. Figure 5 shows that naringenin interacted with the binding pocket formed with amino acid residues of PI3K p110beta (Affinity: −8.5 kcal/mol), PI3K p85alpha (Affinity: −8.0 kcal/mol), AKT3 (Affinity: −7.9 kcal/mol), AKT2 (Affinity: −7.8 kcal/mol), AKT1 (Affinity: −7.7 kcal/mol), or MMP-9 (Affinity: −7.3 kcal/mol). Consequently, the specific molecular interactions between naringenin and PI3K were stronger than those between naringenin and AKT or MMP-9. The 3D image revealed that naringenin bound to PI3K p110beta with several amino acids, including ASP-931, TYR-833, SER-851, VAL-848, and LYS-799 (Figure 5A). Meanwhile, naringenin bound to PI3K p85alpha with ARG-90, ASP-68, and ILE-53 in the active pocket (Figure 5B). Clearly, these sites may represent potential binding sites through which naringenin regulated the function of PI3K proteins.

### 3.6. MD Simulation Analysis of the Interactions Between Naringenin and PI3K Proteins

**Figure 6:**
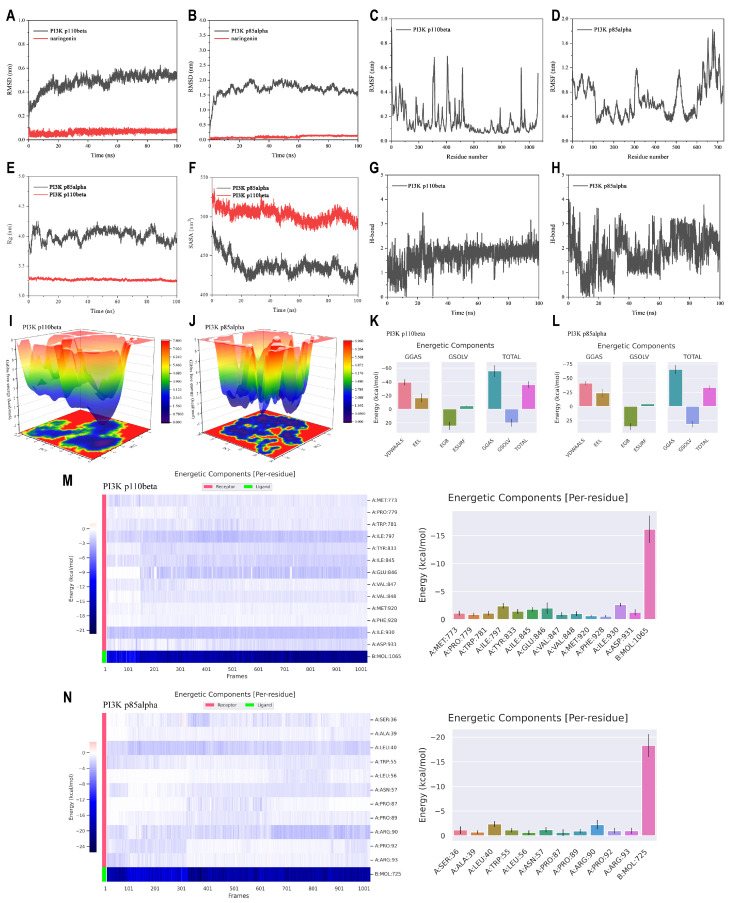
MD simulations of naringenin and PI3K proteins at a 100 ns trajectory. (**A**,**B**) RMSD presentation. (**C**,**D**) RMSF presentation. (**E**) Rg. (**F**) SASA. (**G**,**H**) H-bond. (**I**,**J**) FEL. (**K**,**L**) Free energy calculations from MMPBSA. (**M**,**N**) Energy decomposition analysis of hotspot amino acid residues throughout the trajectory of 100 ns.

To further explore the types of interactions and binding forces needed for the stability between naringenin and PI3K, we performed MD simulations for the naringenin-PI3K p110beta complex and the naringenin-PI3K p85alpha complex. The RMSD models showed the relative stabilities of PI3K p110beta and PI3K p85alpha after the interaction of naringenin with them (Figure 6A,B). The dominant fluctuations of the amino acid residues were observed in PI3K p110beta and PI3K p85alpha as determined in Figure 6C,D. The higher Rg value indicated that PI3K p85alpha had flexible packing and spread-out atomic arrangement throughout stimulation (Figure 6E). Estimation of SASA, a crucial parameter reflecting the extent of the molecule’s surface exposed to the surrounding solvent, was employed to obtain insights into protein conformational alterations following naringenin binding (Figure 6F), which may infer that binding of naringenin to PI3K p110beta and PI3K p85alpha resulted in compression and improved binding. Figure 6G,H indicate that naringenin forms more H-bonds with PI3K p85alpha than with PI3K p110beta. As shown in Figure 6I,J, the naringenin-PI3K p110beta system formed three relatively concentrated large energy basins (3.1~3.9 kcal/mol), whereas the naringenin-PI3K p85alpha complex formed a relatively connected large band of energy basins composed of a dozen small energy basins (3.4~4.2 kcal/mol).

MMPBSA calculations were carried out to determine the molecular interactions (Figure 6K,L). The binding free energies of naringenin with PI3K p110beta and PI3K p85alpha are −35.68 kcal/mol and −33.50 kcal/mol, respectively. These interactions are primarily maintained by van der Waals forces and electrostatic interactions (−39.23 kcal/mol and −16.43 kcal/mol, −41.26 kcal/mol and −23.73 kcal/mol), with polar solvation free energy serving as the main repulsive force. Figure 6M,N display the amino acids ranked by binding energy during MD simulations. Throughout the trajectory of 100 ns, the top three amino acid residues with the highest binding energy in the naringenin-PI3K p110beta complex were ILE-930 (−2.64 kcal/mol), ILE-797 (−2.42 kcal/mol), and GLU-846 (−2.00 kcal/mol). In contrast, LEU-40 (−2.37 kcal/mol) and ARG-90 (−2.25 kcal/mol) consistently contributed significantly to the binding of naringenin in the PI3K p85alpha complex. These results suggested that these amino acids may serve as potential binding sites for naringenin on the PI3K proteins.

### 3.7. Naringenin Binds to PI3K p85alpha and Inhibits the Enzyme Activity of PI3K

**Figure 7:**
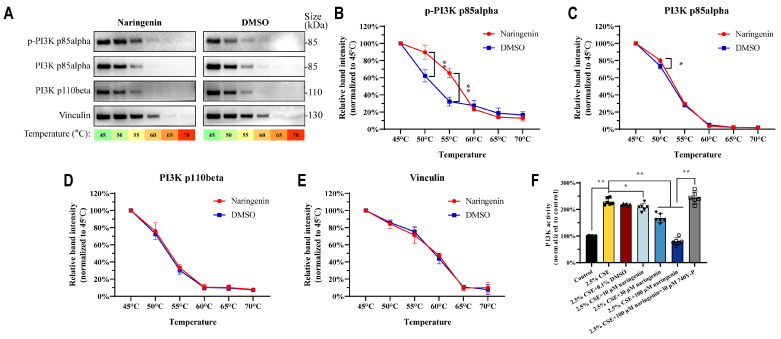
Naringenin could bind with PI3K p85alpha and inhibit PI3K activity in MH-S cells. (**A**) Representative images of PI3K proteins and (**B**–**E**) quantification for the protein levels of (**B**) p-PI3K p85alpha, (**C**) PI3K p85alpha, (**D**) PI3K p110beta, and (**E**) vinculin by CETSA (*n* = 3). (**F**) The effects of naringenin on PI3K enzyme activity in CSE-induced MH-S cells after 24 h treatment (*n* = 6). Data are expressed as mean ± SD. * *p* < 0.05, ** *p* < 0.01.

We present the data for the in silico studies of these ligand receptor complexes wherein PI3K p110beta and PI3K p85alpha were found to have a higher degree of connections than others. The experimental validation was conducted to determine the specific molecular interactions of naringenin and its significance in these molecular systems. As shown in Figure 7A–E, CETSA results showed naringenin significantly improved the protein stability of p-PI3K p85alpha (50 °C) and PI3K p85alpha (50 °C, 55 °C), resulting from drug-target engagement being resistant to thermal denaturation at high temperatures, and this protective effect was not found for PI3K p110beta. Furthermore, naringenin treatment markedly decreased the enzyme activity of PI3K in CSE-induced MH-S cells (Figure 7F). Taken together, CETSA and the enzyme activity assay implied that there were direct interactions between naringenin and PI3K p85alpha, which might contribute to the inhibition of PI3K/AKT pathway activation in MH-S cells.

### 3.8. The Beneficial Effects of Inhibiting MMP-9 Secretion from Alveolar Macrophages Against Elastin and E-Cadherin Damage in Alveolar Epithelial Cells

**Figure 8:**
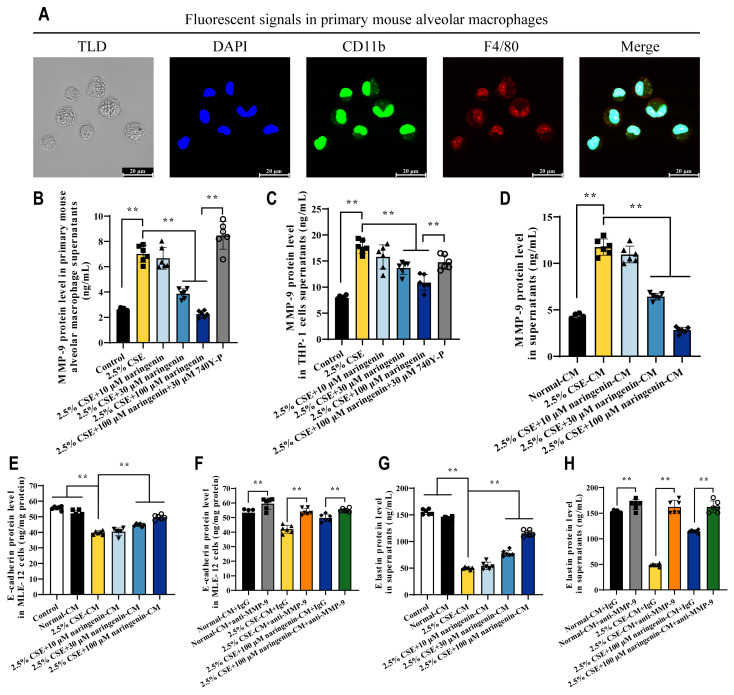
Naringenin protected against ECM injury in alveolar epithelial cells by decreasing the secretion of MMP-9 from alveolar macrophages. (**A**) Confocal microscope images showing the markers in primary mouse alveolar macrophages. From left to right: white light field of TLD, blue DAPI nuclei, green CD11b marker, red F4/80 marker, and three-color fluorescence overlay field. Scale bar at the bottom right corner: 20 μm. (**B**,**C**) The effects of naringenin on the secretion of CSE-induced MMP-9 from mouse primary alveolar macrophages and PMA-induced THP-1 cells with 24 h treatment. (**D**) MMP-9 levels in CM from drug removal MH-S cells for 12 h. (**E**–**H**) The effects of CM and MMP-9-inhibited CM on E-cadherin and elastin at 24 h. Data are expressed as mean ± SD (*n* = 6). ** *p* < 0.01.

To further clarify the inhibitory effect of naringenin on MMP-9 secretion by alveolar macrophages, we isolated primary mouse alveolar macrophages from the BALF of C57BL/6 mice, which were used to validate the efficacy of naringenin in inhibiting CSE-induced MMP-9 secretion, and experiments were also conducted using human THP-1-differentiated macrophages. Figure 8A indicates that the isolated cells from BALF expressed both CD11b and F4/80 markers, confirming them as mature macrophages from alveoli suitable for the next experiments. Consistent with the results in MH-S cells, 2.5% CSE significantly increased MMP-9 secretion in both (Figure 8B) primary mouse alveolar macrophages and (Figure 8C) human THP-1-differentiated macrophages, and naringenin at concentrations of 30 μM and 100 μM significantly inhibited CSE-induced MMP-9 secretion, while this inhibitory effect of naringenin was diminished by 740Y-P.

We designed an experiment to investigate whether MMP-9 released by alveolar macrophages in response to CSE and naringenin affected alveolar epithelial ECM. After co-treating MH-S cells with 2.5% CSE and varying concentrations of naringenin for 24 h, the MMP-9 levels in the culture medium remained consistent in their differential trends even 12 h post-removal of CSE and naringenin (Figure 1B and Figure 8D). Accordingly, the 12 h treatment CM from drug removal MH-S cells can be used to study the effects of MMP-9 secretion by alveolar macrophages on alveolar epithelial cells. Figure 8E,G suggest a negative correlation between MMP-9 levels of CM and the degradation of E-cadherin and elastin in MLE-12 cells. Figure 8F,H indicate that compared to the IgG treatment group, inhibiting MMP-9 activation using the anti-MMP-9 antibody significantly increased the levels of E-cadherin and elastin in MLE-12 cells. Therefore, naringenin not only inhibited MMP-9 secretion from alveolar macrophages but also could offer protective effects against damage to alveolar epithelial E-cadherin and elastin.

## 4. Discussion

CSE-induced cell models are widely employed for studying the molecular mechanisms in cigarette smoke-induced lung diseases [42]. The increase in MMP-9 in the cigarette smoke-exposed lungs promotes ECM degradation of bronchi and alveoli [43], leading to the disruption of the epithelial basement membrane, thereby exacerbating inflammatory responses in the airways [44]. Interventions that inhibit MMP-9 secretion from alveolar macrophages may represent a promising therapeutic approach for cigarette smoke-induced lung injury [45]. Research indicated that alveolar macrophages from smokers released higher levels of MMP-9 [46]. Cigarette smoke exposure significantly elevated the expression and activity of MMP-9 in the mouse lungs [47], and in vitro experiments showed that CSE-stimulated alveolar macrophages exhibited markedly elevated levels of MMP-9 expression and secretion [48,49]. These results were consistent with the evidence we provided here. Our results indicated that CSE-induced MH-S cells exhibit a significant increase in MMP-9 expression and secretion, which can be inhibited by naringenin. Furthermore, the inhibitory effect of naringenin on CSE-induced MMP-9 secretion was also validated in primary mouse alveolar macrophages and THP-1-differentiated macrophages, suggesting that naringenin has potential therapeutic effects in alleviating cigarette-stimulated pulmonary lesions.

Numerous studies have indicated that the PI3K/AKT signaling pathway is related to the expression of MMP-9 [50]. Previous reports had confirmed that MMP-9 was released from RAW264.7 macrophages via the PI3K/AKT axis [51]. Naringenin was shown to downregulate the PI3K/AKT signaling pathway [52], subsequently inhibiting MMP-9 expression [30]. However, the effects of naringenin on mediating MMP-9 expression and secretion in cigarette smoke-exposed alveolar macrophages remain underexplored. Our findings demonstrated that naringenin inhibited CSE-induced upregulation of the PI3K/AKT signaling pathway, leading to the decreased expression and secretion of MMP-9 in alveolar macrophages. Notably, the inhibitory effect of naringenin can be reversed by the PI3K activator 740Y-P.

Our study elucidated the target of naringenin in the PI3K/AKT signaling pathway. Molecular docking results indicated that naringenin interacted more strongly with PI3K than with AKT, and MD simulations studied naringenin’s interactions with PI3K p110beta and PI3K p85alpha. CETSA showed that naringenin was directly combined with PI3K p85alpha and its phosphorylated form, rather than with PI3K p110beta. Moreover, the enzyme activity assay confirmed that naringenin inhibited CSE-induced increased activity of PI3K. The information collected from these techniques was demonstrated to provide conformational perspectives, clarify the binding system between the enzyme and the ligand, and corroborate the experimental results. Generally, our results suggested that the inhibitory effect of naringenin on MMP-9 in alveolar macrophages was PI3K p85alpha-dependent, binding with the active amino acid residues ARG-90, ASP-68, ILE-53, and LEU-40.

Communications between alveolar macrophages and epithelial cells are crucial for maintaining pulmonary homeostasis and play an important role in cigarette smoke-induced lung injury [53]. MMP-9 modulates the bioactive molecules of ECM by directly cleaving elastin, type IV collagen, fibrillin, and other components [54]. Loss of E-cadherin in alveolar epithelial type II cells resulted in airspace enlargement and damage of intercellular adhesion, accelerating early pathological changes in pulmonary epithelium [55]. Additionally, the loss of elastin within alveolar attachments led to terminal bronchiole collapse, lumen narrowing, and airflow obstruction [56]. Here, in vitro intervention experiments indicated that inhibiting MMP-9 derived from alveolar macrophages reduced the degradation of alveolar epithelial E-cadherin and elastin, thereby providing circumstantial protective effects of naringenin against alveolar damage.

Multitudinous in vivo studies have confirmed that smoking increases MMP-9 levels in the lungs, and naringenin can inhibit the expression of MMP-9 in mouse BALF, lung tissue, and serum [57]. Although we confirmed that naringenin suppressed MMP-9 expression and secretion in MH-S cells, THP-1 cells, and primary mouse alveolar macrophages, a limitation of our study is the lack of validation using in vivo models. Therefore, conducting intensive insights on naringenin’s targeting of alveolar macrophages in in vivo models is an important next step.

## 5. Conclusions

**Figure 9:**
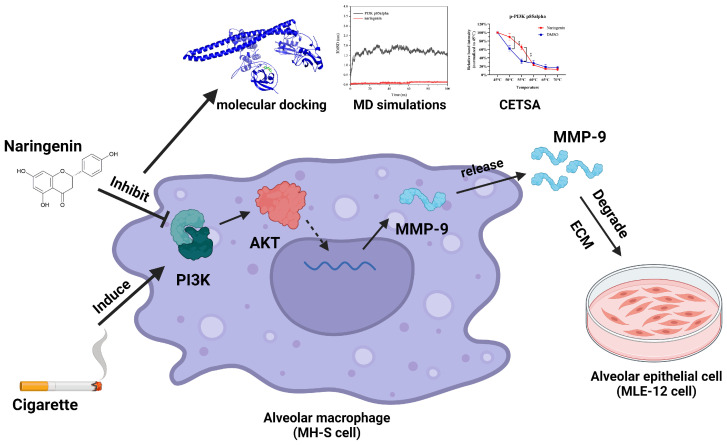
Schematic diagram illustrating naringenin’s effects on the PI3K/AKT/MMP-9 pathway in CSE-induced alveolar macrophages, ** *p* < 0.01.

Our study provided a targeted evaluation of naringenin’s regulatory effects on MMP-9 through multi-techniques encompassing ELISA, proteomics analysis, Western blot, molecular docking, MD simulations, CETSA, and enzyme assay. Naringenin may act as a PI3K targeted inhibitor, controlling the PI3K/AKT signaling pathway and curbing the expression of MMP-9 in CSE-induced alveolar macrophages (Figure 9). It is also protected against ECM protein (elastin and E-cadherin) injury in alveolar epithelial cells by decreasing the secretion of MMP-9 from alveolar macrophages. Our findings on naringenin’s inhibitory effects provided valuable insights that could lead to developing naringenin treatment for cigarette smoke-induced lung injury.

## Data Availability

The proteomics data have been deposited to the ProteomeXchange Consortium (https://proteomecentral.proteomexchange.org, accessed on 24 April 2025) via the iProX partner repository with the dataset identifier PXD063137. The data generated during this study are presented in the manuscript and are available from the corresponding author on reasonable request.

## Abbreviations

The following abbreviations are used in this manuscript:

BALF	bronchoalveolar lavage fluid
BCA	bicinchoninic acid assay
CETSA	cellular thermal shift assay
CM	conditioned medium
COPD	chronic obstructive pulmonary disease
CSE	cigarette smoke extract
DMSO	dimethyl sulfoxide
ECM	extracellular matrix
ELISA	enzyme linked immunosorbent assay
FC	fold change
FDR	false discovery rate
FEL	free energy landscape
H-bond	hydrogen bond
LC	liquid chromatography
MD simulations	molecular dynamics simulations
MMPBSA	molecular mechanics Poisson–Boltzmann surface area
MMP-9	matrix metalloproteinase-9
MS	mass spectrometer
MTS	3-(4,5-Dimethylthiazol-2-yl)-5-(3-carboxymethoxyphenyl)-2-(4-sulfophenyl)-2H-tetrazolium
NPT	constant-pressure and constant-temperature
NVT	canonical ensemble
Rg	radius of gyration
RMSD	root mean square deviation
RMSF	root mean square fluctuation
SASA	solvent accessible surface area
SD	standard deviation
